# Substrate Reduction Augments the Efficacy of Enzyme Therapy in a Mouse Model of Fabry Disease

**DOI:** 10.1371/journal.pone.0015033

**Published:** 2010-11-24

**Authors:** John Marshall, Karen M. Ashe, Dinesh Bangari, KerryAnne McEachern, Wei-Lien Chuang, Joshua Pacheco, Diane P. Copeland, Robert J. Desnick, James A. Shayman, Ronald K. Scheule, Seng H. Cheng

**Affiliations:** 1 Applied Discovery Research, Genzyme Corporation, Framingham, Massachusetts, United States of America; 2 Genetics and Genomic Sciences, Mount Sinai School of Medicine, New York, New York, United States of America; 3 Internal Medicine – Nephrology, Genzyme Corporation, University of Michigan, Ann Arbor, Michigan, United States of America; National Institutes of Health, United States of America

## Abstract

Fabry disease is an X-linked glycosphingolipid storage disorder caused by a deficiency in the activity of the lysosomal hydrolase α-galactosidase A (α-gal). This deficiency results in accumulation of the glycosphingolipid globotriaosylceramide (GL-3) in lysosomes. Endothelial cell storage of GL-3 frequently leads to kidney dysfunction, cardiac and cerebrovascular disease. The current treatment for Fabry disease is through infusions of recombinant α-gal (enzyme-replacement therapy; ERT). Although ERT can markedly reduce the lysosomal burden of GL-3 in endothelial cells, variability is seen in the clearance from several other cell types. This suggests that alternative and adjuvant therapies may be desirable. Use of glucosylceramide synthase inhibitors to abate the biosynthesis of glycosphingolipids (substrate reduction therapy, SRT) has been shown to be effective at reducing substrate levels in the related glycosphingolipidosis, Gaucher disease. Here, we show that such an inhibitor (eliglustat tartrate, Genz-112638) was effective at lowering GL-3 accumulation in a mouse model of Fabry disease. Relative efficacy of SRT and ERT at reducing GL-3 levels in Fabry mouse tissues differed with SRT being more effective in the kidney, and ERT more efficacious in the heart and liver. Combination therapy with ERT and SRT provided the most complete clearance of GL-3 from all the tissues. Furthermore, treatment normalized urine volume and uromodulin levels and significantly delayed the loss of a nociceptive response. The differential efficacies of SRT and ERT in the different tissues indicate that the combination approach is both additive and complementary suggesting the possibility of an improved therapeutic paradigm in the management of Fabry disease.

## Introduction

The lysosomal storage disorder (LSD) Fabry disease is caused by mutations in the gene encoding the lysosomal hydrolase α-galactosidase A (α-gal) [1]. Deficiency in α-gal activity results in the abnormal accumulation of neutral glycosphingolipids, in particular globotriaosylceramide (GL-3) in many cell types. Vascular endothelium accumulation plays a major role, leading to kidney dysfunction, cardiac and cerebrovascular disease [2]. The current standard of care for Fabry patients is enzyme-replacement therapy (ERT) through periodic infusions of recombinant human α-gal (agalsidase beta or agalsidase alpha). This treatment has been shown to be effective at slowing the loss of renal function [3], [4] and at reducing the cardiac disease [5]. However, its ability to abate disease progression particularly in patients with more advanced manifestations is more modest [6], [7]. Also, the degree of accumulated GL-3 clearance varies depending upon the cell-type [8]. Hence, alternative or adjuvant therapies may provide an improvement over the existing treatment paradigm.

Several alternative therapeutic options have been evaluated for LSDs [9], with substrate reduction therapy (SRT) being the most promising based on its demonstrated efficacy in Gaucher disease and oral availability [10], [11]. SRT works on the principle of limiting the production of the pathologic substrate, which in the case of Fabry disease is primarily GL-3. This can be achieved by inhibiting the enzyme glucosylceramide synthase which catalyzes the first step in the synthesis of glycosphingolipids (GL-1) and therefore subsequent molecules including GL-3. The premise of SRT for Fabry disease using inhibitors of glucosylceramide synthase has been evaluated in mouse models [12]–[14] and shown to be of some benefit in lessening the burden of glycolipid accumulation. A candidate inhibitor, miglustat, is approved for treatment of type 1 Gaucher disease (in patients for whom ERT is not a therapeutic option). It is however associated with adverse side effects [15], [16] that may compound reported Fabry disease symptoms (*e.g.* diarrhea, peripheral neuropathy) and is not approved for this indication [17]. It is desirable to have alternative small molecule drugs with the appropriate safety profile for SRT of Fabry disease. However, it should be noted that because the majority of Fabry patients are null for α-gal activity, SRT as a monotherapy is unlikely to be as effective as it has been shown for type 1 Gaucher patients, whom invariably retain some residual glucocerebrosidase activity. This distinction suggests that a combination of ERT and SRT may be a more beneficial approach to managing Fabry disease.

To evaluate the relative merits of a combined SRT and ERT approach for Fabry disease, we utilized a drug already shown to be active in SRT for Gaucher disease [11], namely eliglustat tartrate (Genz-112638). As an inhibitor of glucosylceramide synthase, eliglustat tartrate has been shown to be effective as both a monotherapy and in combination with ERT in a mouse model of Gaucher disease [18], [19]. In the current studies, we evaluated this molecule in an α-galactosidase A knockout mouse [20] that exhibits many similarities with the human disease. Although the disease manifestations are less severe than noted in human, the mouse models of Fabry disease [20], [21] nevertheless can provide valuable information on the merits of various therapeutic interventions [22]. Evaluations of potential therapies in these mice have focused mainly on tissue levels of GL-3, but other disease relevant symptoms could also be measured, such as heat-sensitivity as a marker of peripheral neuropathy [23], [24]. Here, we describe our evaluation of SRT (using eliglustat tartrate) as a monotherapy as well as SRT in combination with ERT (using recombinant human α-galactosidase A) in a Fabry mouse model. We demonstrate that the therapeutic benefit of the combined therapy approach is both additive and complementary for treating this disease.

## Results

The majority of our studies with SRT using eliglustat tartrate were performed using immunocompetent Fabry mice. However, because infusions of α-gal elicited the production of antibodies against the recombinant human enzyme (that may complicate interpretation of the results), studies that involved the use of ERT were performed using the Fabry-Rag mouse model, which does not generate a humoral response to recombinant human α-gal (data not shown). Although multiple symptoms are apparent in patients, Fabry mouse models are relatively normal except for modest accumulation of GL-3. We have noted previously [23] that the Fabry mouse also displays a progressive functional deficit in heat sensitivity as well as pathology in the dorsal root ganglia. In addition to monitoring this sensory readout, we also measured renal parameters to monitor the potential impact of treatment on kidney function.

### SRT reduces the rate of GL-3 accumulation in visceral tissues

**Figure 1:**
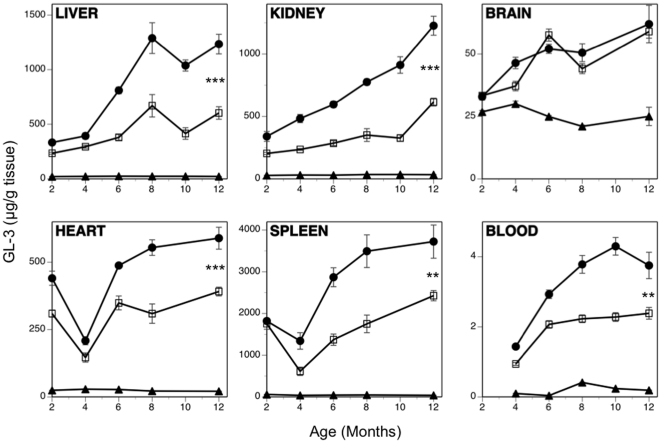
Substrate reduction therapy delays the accumulation of GL-3 in visceral tissues of Fabry mice. Treated Fabry mice received eliglustat tartrate as a component of their food beginning at 5 weeks of age. The GL-3 levels in various tissues of untreated control Fabry (•), eliglustat tartrate-treated Fabry (□) and wild-type mice (▴) were determined using a mass spectrometry assay and are presented as µg GL-3/g wet tissue. Data are shown as mean ± SEM (n = 5 mice/time point). Statistics were performed using the Graphpad Prism software t test (** = p<0.01; *** = p<0.001).

One-month old Fabry mice were either untreated or dosed with eliglustat tartrate (in food) and sacrificed over time to quantify tissue GL-3 levels. **Figure 1** shows that in untreated mice, all the visceral tissues evaluated (liver, kidney, heart and spleen) as well as whole blood, accumulated GL-3 over the 11 month course of the study to levels at least 100-fold higher than wild-type control GL-3 levels. Treated Fabry mice also continued to accumulate GL-3 throughout the course of the study, but did so at a reduced rate, generally resulting in significant net GL-3 reductions of 40–50% by the end of the study. One exception was the brain, where no difference was seen between treated and untreated mice, though the increase of GL-3 in both treated and untreated Fabry mice was significant (p<0.0001) relative to the wild-type control. This lack of treatment efficacy in the CNS was expected, because eliglustat tartrate is a substrate for P-glycoprotein [11] and as such fails to distribute to the brain parenchyma effectively. Overall, these results are consistent with an ability of eliglustat tartrate to inhibit (to some degree) the synthesis of glycosphingolipids such as GL-3, but because this inhibition is not complete and given the total absence of α-gal in the model, these glycosphingolipids continue to accumulate in most tissues albeit at a significantly reduced rate.

### SRT normalizes urine volume and decreases urine GL-3 levels

**Figure 2:**
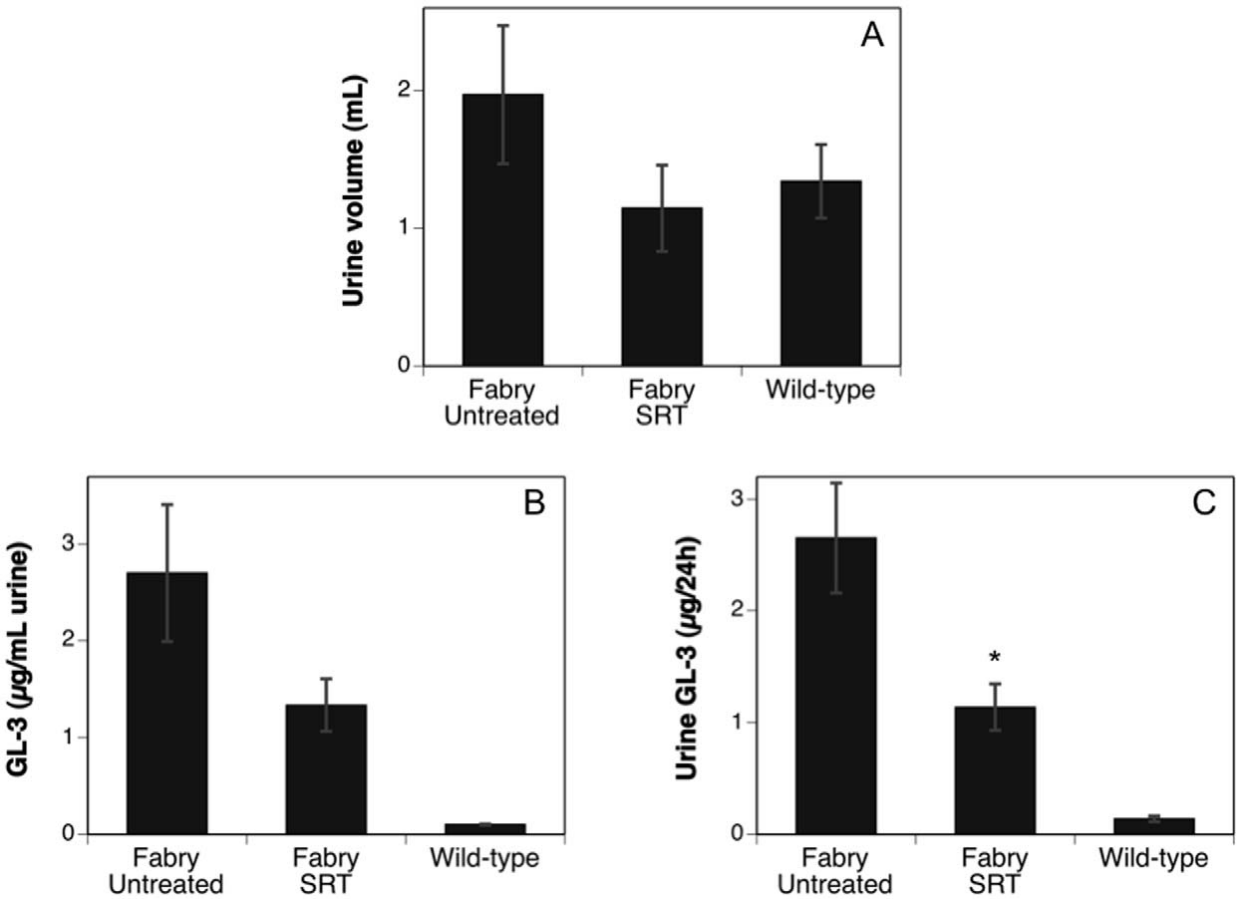
Substrate reduction therapy normalizes urine volume and decreases urine GL-3 levels. Treated Fabry mice received eliglustat tartrate as a component of their food beginning at 5 weeks of age. Urine was collected from eliglustat tartrate (SRT)-treated or untreated Fabry mice and also from age-matched wild-type mice (11-months of age) housed in metabolic cages for 24 h. (**A**) Total urine volume. (**B**) Concentration of GL-3 in the urine. (**C**) Total GL-3 excreted in urine over 24 h. Data are shown as mean ± SEM (n = 15 mice/group). Statistics were determined using the Graphpad Prism software t test (* = p<0.05 compared to Fabry untreated).

In untreated Fabry patients, GL-3 deposition in the kidney leads to proteinuria and progressively decreasing glomerular filtration rates. Later stage renal dysfunction then leads to uremia and is a common cause of death [2]. Initial renal symptoms, however, may include polyuria due to a concentration defect [25]. Enzyme replacement therapy can successfully ameliorate these symptoms, especially in patients with early-stage renal insufficiency [26]. As a model, the Fabry mouse appears to recapitulate early, rather than later effects of the disease process. Thus, for example, there was no evidence of renal failure in 80-week old mice in one Fabry mouse model [27]. Consistent with this view, a screening of renal parameters in our mouse model revealed increases in urine volume and urine GL-3 that could be used as biomarkers related to the disease process. **Figure 2A** shows that the volume of urine produced by the cohort of untreated Fabry mice trended toward a higher level relative to that produced by wild-type mice; SRT normalized urine volume to wild-type levels. **Figure 2B** shows that there was an ∼20-fold increase in urine GL-3 levels in the untreated Fabry mice relative to that of age-matched wild-type controls. Treating Fabry mice with eliglustat tartrate (SRT) reduced the urine GL-3 concentration to about 50% that of the untreated mice, mirroring the benefit observed in the visceral tissues (Figure 1). It should be noted that the carbon chain length and form of the urine GL-3 acyl groups more closely matched those detected in the mouse kidney profile and differed significantly from that of the blood (data not shown). This suggests that the source of the urine GL-3 is not a representative blood filtrate but more likely derived from the kidney. **Figure 2C** depicts the total GL-3 excreted over 24 h, showing that although SRT significantly decreased urine GL-3 (∼2.5-fold relative to untreated Fabry mice), it remained ∼10-fold higher than wild-type levels. Together with the data in Figure 1 showing GL-3 deposition in the kidney over time, these results demonstrate renal involvement in the Fabry mouse with physiologic consequences that may mirror the early stages of kidney pathophysiology in Fabry patients. They also suggest that SRT may provide benefit in terms of delaying the loss of renal function.

### SRT delays the onset of heat-insensitivity in Fabry mice

**Figure 3:**
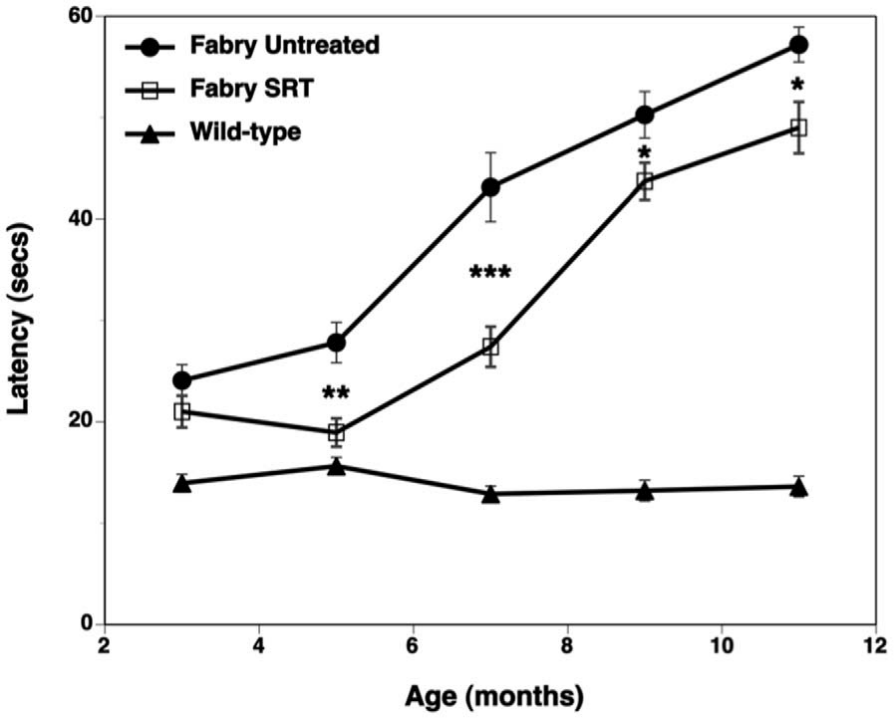
Substrate reduction therapy delays the onset of heat-insensitivity in Fabry mice. Treated Fabry mice received eliglustat tartrate as a component of their food beginning at 5 weeks of age. Mice were placed on a 55°C hot-plate and the time for them to respond (latency) recorded. Latency was measured every two months from 3-months of age for untreated control Fabry (•), eliglustat tartrate treated Fabry (□), and wild-type mice (▴). Statistics compare the treated to the untreated Fabry mice. Data are shown as mean ± SEM (n = 15 mice/time point). Statistics were determined using the Graphpad Prism software t test (* = p<0.05; ** = p<0.01; *** = p<0.001).

Fabry disease is characterized by small fiber dysfunction in both male and female patients that results in increased thresholds for both heat and cold stimuli [28]–[31]. Consistent with this small fiber neuropathy, Fabry mice also develop a demonstrable deficit in their ability to respond to a heat stimulus [22], [23]. Potential therapeutics can be evaluated in this model for their effects on a nociceptive response to a heat stimulus by measuring the time taken by the mouse to react (latency) after being placed on a 55°C hot-plate. **Figure 3** shows that there was no difference in latency between treated and untreated Fabry mice at 3 months of age, although both groups reacted more slowly to heat than the wild-type mice at this initial time point. Wild-type mice showed no change in latency throughout the study. However, consistent with our earlier report [23], untreated Fabry mice developed a progressive thermal hypoalgesia beginning at 5 months of age. An equivalent deficit was not observed in the eliglustat tartrate-treated Fabry mice until 7 months of age. This two-month delay in the progression of loss of heat-sensitivity between the treated and untreated Fabry mice was consistent and significant throughout the course of the study. Collectively, these results in the Fabry mouse suggest that SRT with eliglustat tartrate can provide benefit by delaying GL-3 accumulation and its attendant (renal and neurologic) symptoms. Because the Fabry mouse is null for α-gal, we reasoned that stabilization or actual reduction of the GL-3 burden would be more likely if recombinant α-gal were included in the treatment regime. To evaluate this possibility, we chose to use the Fabry-Rag model (these mice do not develop mature B or T cells), thereby eliminating any humoral immune response to α-gal epitopes that might complicate interpretation of the experimental results.

### Combined ERT and SRT is most effective at reducing GL-3 levels in tissues of the Fabry-Rag mouse

The combination of ERT and SRT was evaluated in 3-month old Fabry-Rag mice, an age at which significant GL-3 has accumulated; absolute GL-3 levels were comparable between Fabry and Fabry-Rag mice (data not shown). Fabry mice were treated either by ERT or SRT alone or by combination SRT and ERT. For the monotherapy studies, Fabry mice were administered either 300 mg/kg/day eliglustat tartrate in the food throughout the course of the study (SRT) or α-gal (1 mg/kg) every 2 months (ERT2). For the combination studies, animals received 300 mg/kg/day eliglustat tartrate and α-gal (1 mg/kg) dosed IV every 2 months (ERT2+SRT) or 4 months (ERT4+SRT). The latter was designed to determine whether the frequency of enzyme treatments could be reduced when combined with SRT. Mice were killed beginning at 3-months of age, and tissue GL-3 levels determined. Visceral tissues (liver, kidney, heart and spleen), whole blood and urine all demonstrated elevated levels of GL-3 in the Fabry-Rag mouse relative to the wild-type controls at all time points (data not shown).

**Figure 4:**
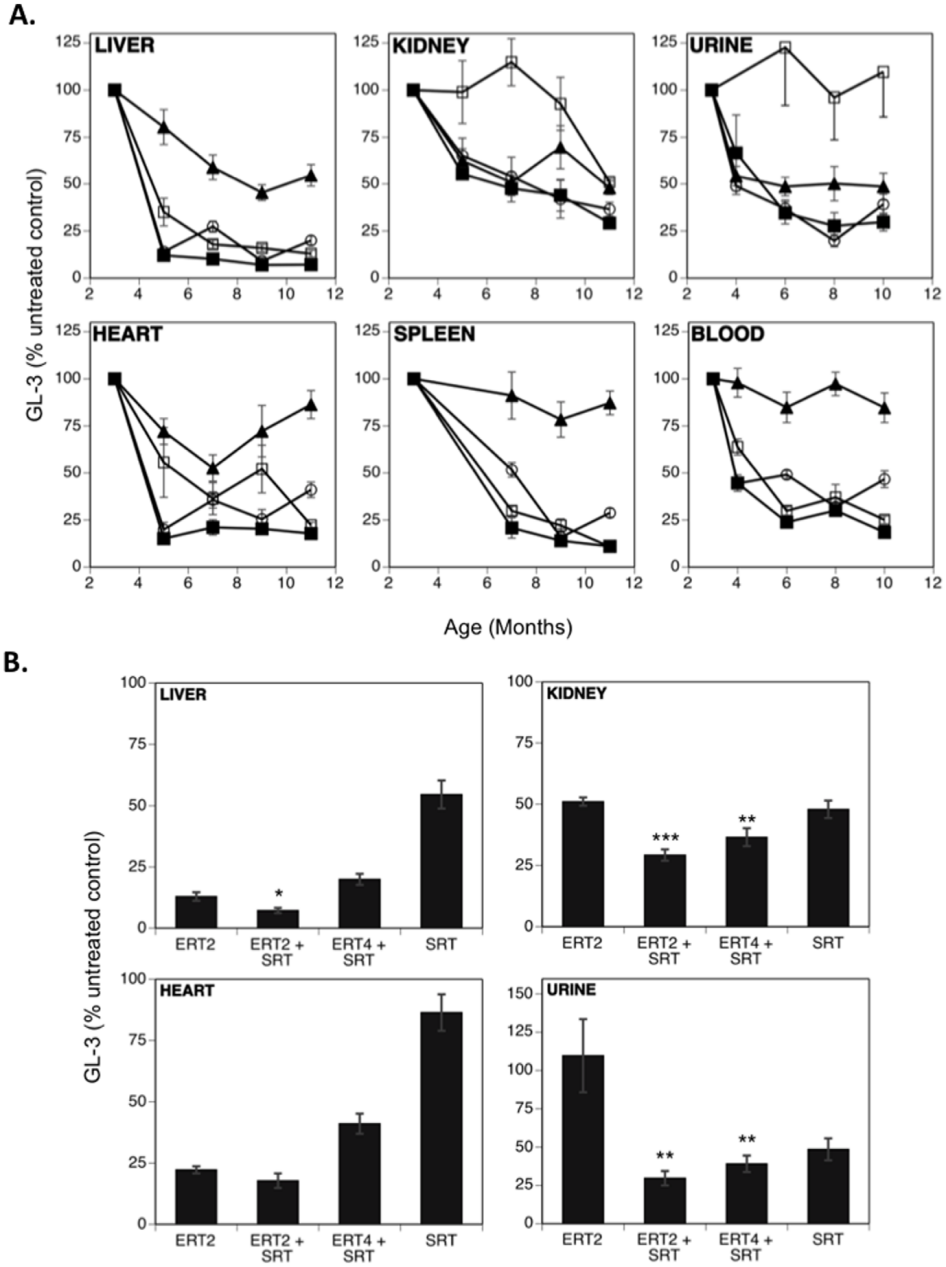
Combined enzyme-replacement therapy and substrate reduction therapy is most effective at reducing GL-3 levels in visceral tissues and fluids of the Fabry-Rag mouse. Untreated age-matched Fabry-Rag mice were used to standardize the GL-3 levels. (**A**) GL-3 levels over time for groups treated with ERT every two months (**□**),ERT every two months+eliglustat tartrate (▪), ERT every four months+eliglustat tartrate (**○**) and eliglustat tartrate alone (▴). (**B**) GL-3 levels at the end of the study in liver, kidney, heart (all 11-months old) and urine (10-months old) for groups treated with ERT every two months (ERT2), ERT every two months+eliglustat tartrate (ERT2+SRT), ERT every four months+eliglustat tartrate (ERT4+SRT) and eliglustat tartrate alone (SRT). Statistical comparisons are to the ERT2 treatment group. Data are shown as mean ± SEM (n = 5 mice/time point except 11-month tissue and all blood and urine samples where n = 10 mice/time point). Statistics were determined using the Graphpad Prism software t test (* = p<0.05; ** = p<0.01; *** = p<0.001).

**Figure 4A** shows that although all treatment paradigms reduced GL-3, the greatest and most consistent reductions regardless of tissue were obtained using infusions of α-gal every two months plus eliglustat tartrate. **Figure 4B** shows the results for liver, kidney, heart and urine at the end of the study (11 months of age). In the liver and heart, ERT was the more effective monotherapy strategy, with additional benefit realized in the liver when SRT was deployed in combination. In the kidney, ERT and SRT alone were equally effective, but their combination was significantly more effective. Urine GL-3 was effectively reduced by SRT, with some added benefit by ERT. The kidney and urine both showed highly significant improvements in GL-3 levels with either regimen of combination therapy (ERT2 or ERT4) when compared to the ERT alone group. This observation suggests that a combination of ERT and SRT may benefit those Fabry patients whose renal function continues to decline even when on ERT [26]. Taken together, these results suggest that in all the tissues tested, maximum GL-3 reduction can be obtained using a combination of ERT plus SRT, and that within this context, more frequent dosing with enzyme appears to be more effective.

### Combined ERT and SRT is most effective at reducing uromodulin levels in the urine of Fabry-Rag mice

**Figure 5:**
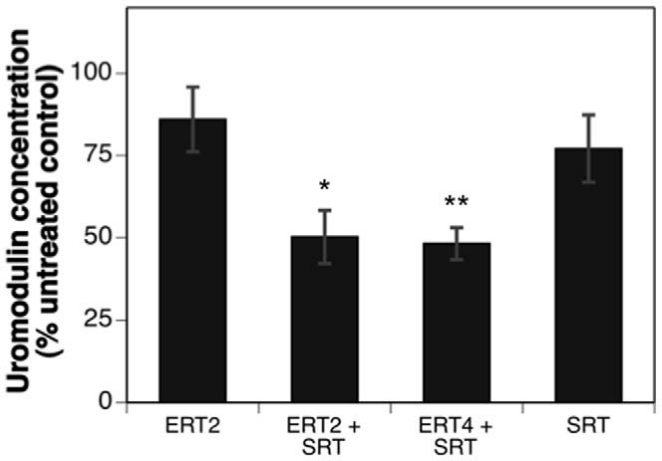
Combined enzyme-replacement therapy and substrate reduction therapy is most effective at reducing uromodulin levels in the urine of Fabry-Rag mouse. Urine was collected from 10-month old Fabry-Rag mice. Mice receiving no treatment were used to standardize urine volume and uromodulin levels for those treated with ERT every two months (ERT2), ERT every two months+eliglustat tartrate (ERT2+SRT), ERT every four months+eliglustat tartrate (ERT4+SRT) and eliglustat tartrate alone (SRT). Relative urine uromodulin levels from the Fabry-Rag mice were determined using a sandwich ELISA. Data are presented as % of uromodulin concentration relative to an age-matched untreated control Fabry-Rag mouse. Data are shown as mean ± SEM (n = 10 mice/time point). Statistical comparisons are to the ERT2 treatment group and were determined using the Graphpad Prism software t test (* = p<0.05; ** = p<0.01; *** = p<0.001).

Uromodulin (Tamm-Horsfall glycoprotein) is the most abundant protein in normal mammalian urine. It resides as a glycophosphatidylinositol-anchored protein on the tubular epithelial cells of the ascending loop of Henle from where it is proteolytically cleaved and excreted. Uromodulin expression and processing appears to be abnormal in Fabry patients [32]. Characterization of the Fabry-Rag mouse showed that urine uromodulin levels were significantly elevated. **Figure 5** shows that although monotherapy by SRT and ERT had some ability to decrease urine uromodulin concentrations (20–25% reduction compared to untreated), combining ERT and SRT appeared to result in an additive effect, reducing uromodulin concentrations by at least 50%. Taking into account the effects of combination ERT and SRT on urine volume (data not shown; see Figure 2), total uromodulin amounts excreted over a 24 h period were most effectively reduced by the combined ERT and SRT approaches (∼75% compared to untreated; data not shown).

In untreated Fabry patients, uromodulin excretion has been reported as being reduced [32], while we found an increase in the untreated Fabry-Rag mouse model. In addition to possible differences in the extent of kidney pathology between the mouse model and patients, it is worth noting that differences in ELISA capture antibodies together with post-translational processing of uromodulin [32] may help account for these apparent discrepancies. In any case, excreted uromodulin may be reflecting GL-3 buildup in the distal tubules, and these changes can be normalized most effectively in the mouse model by a combined ERT plus SRT approach.

### SRT is most effective at delaying the onset of heat-insensitivity in Fabry-Rag mice

**Figure 6:**
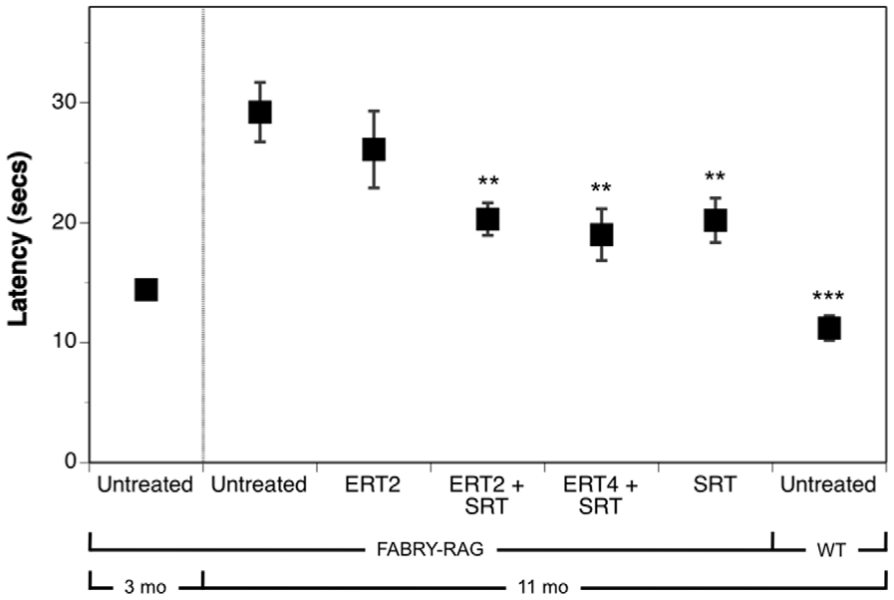
Substrate reduction therapy is most effective at delaying the onset of heat-insensitivity in Fabry-Rag mice. Treatments were begun at 3 months of age. Latency in treated Fabry-Rag mice and in an age-matched wild-type control group was measured at 11 months of age; a 3-month old untreated Fabry-Rag cohort was included for comparison. Mice were placed on a 55°C hot-plate and the time for them to respond (latency) recorded. Latency is shown for wild-type mice, Fabry-Rag mice treated with ERT every two months (ERT2), ERT every two months+eliglustat tartrate (ERT2+SRT), ERT every four months+eliglustat tartrate (ERT4+SRT), eliglustat tartrate alone (SRT) and an untreated Fabry-Rag cohort. Data are shown as mean ± SEM (n = 10 mice/time point). Statistical comparisons are to the untreated Fabry-Rag mice at 11 months of age, and were determined using the Graphpad Prism software t test (* = p<0.05; ** = p<0.01; *** = p<0.001).

The effects of the different therapeutic regimes on thermal sensitivity in Fabry-Rag mice were also evaluated using the hot-plate assay (Figure 3). **Figure 6** shows that the latency of Fabry-Rag mice at 3 months of age (start of study) to react upon being placed on a hot-plate was already significantly increased relative to that of wild-type mice that were 11 months of age (end of study). Left untreated, the latency of Fabry-Rag mice to react to the hot-plate essentially doubled over the eight months of the experiment. Although this progressive increase in latency in the Fabry-Rag was qualitatively similar to that observed in the Fabry model (Figure 3), it was not as dramatic, and did not approach the latency (60 sec) seen in the immunocompetent Fabry mouse. Consistent with the wild-type result shown in Figure 3, latency in the wild-type mouse cohort was unchanged over this same time frame (data not shown). Fabry-Rag mice treated with ERT alone showed no improvement in response time compared to untreated controls. Mice that received SRT alone or SRT combined with ERT showed similarly reduced response times and were all significantly more sensitive to the heat stimulus than untreated Fabry-Rag controls. Taken together, these data suggest that SRT is more effective than ERT at delaying the loss of heat sensitivity in the Fabry-Rag mouse and may therefore provide benefit in treating the peripheral nervous system related symptoms of Fabry patients.

### Combined ERT and SRT is most effective at reducing the number of vacuolated neurons in the dorsal root ganglia of Fabry-Rag mice

**Figure 7:**
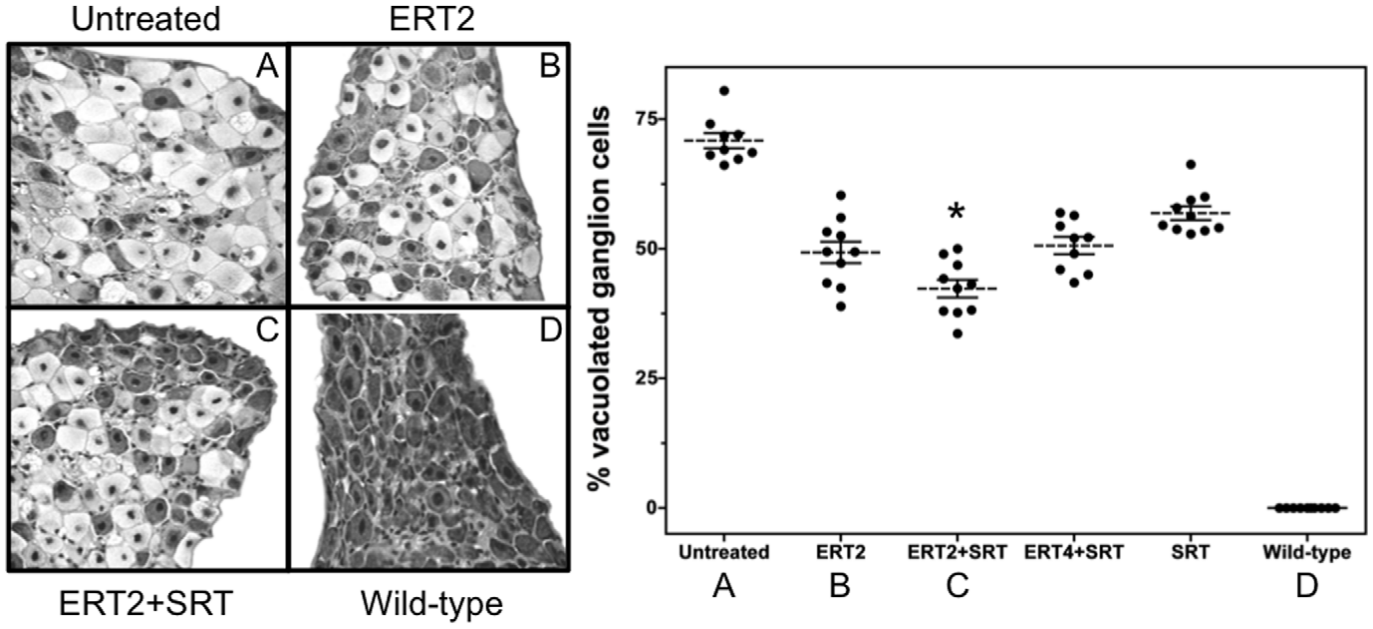
Combined enzyme-replacement therapy and substrate reduction therapy is most effective at reducing the number of vacuolated ganglion cells in DRGs of the Fabry-Rag mouse. Spinal cords from 11-month old mice were processed for hematoxylin and eosin staining. Photomicrographs (400×) of the dorsal root ganglia were taken to highlight the degree of vacuolation in the ganglion cells of the thoracic section. (**Left Panel**) Representative images are shown for (**A**) untreated Fabry-Rag mice, (**B**) Fabry-Rag mice treated with ERT every two months only (ERT2), (**C**) Fabry-Rag mice treated with ERT every two months and SRT (ERT2+SRT), and (**D**) wild-type controls. (**Right Panel**) Multiple sections were examined microscopically and the percent vacuolated DRG cells in each section determined. Groups of Fabry-Rag mice were treated with ERT every two months (ERT2), ERT every two months+eliglustat tartrate (ERT2+SRT), ERT every four months+eliglustat tartrate (ERT4+SRT), eliglustat tartrate alone (SRT). Age-matched untreated Fabry-Rag and wild-type mice were included as controls. Data are shown as individual scores (•), mean (dashed line) ± SEM (n = 10 mice/time point). Statistics were determined using the Graphpad Prism software t test (* = p<0.05 compared to ERT2 treated).

Cell bodies of primary sensory neurons are located in the dorsal root ganglia (DRG); their afferent axons project into the spinal column. The neuropathic pain characteristic of Fabry disease may have its origin in the accumulation of GL-3 in DRG neurons and their subsequent destruction [33]. We had previously shown that at nine months of age, the DRG cell bodies of Fabry mice appear vacuolated and distended, and that this vacuolation could be partially prevented by providing exogenous α-gal from a gene therapy vector [23]. We therefore monitored the DRG morphology to assess the relative efficacies of SRT and ERT in the Fabry-Rag model. Vacuolated DRG neurons were quantified after 8 months of treatment. Spinal cords were processed for H&E staining to reveal the presence of vacuolated cells in the dorsal root ganglia. **Figure 7** (left panel) shows qualitatively that the untreated Fabry-Rag DRGs had the most severe vacuolation (lighter shaded cell bodies), consistent with our previous findings in the immunocompetent Fabry model. Treatment with ERT alone appeared to decrease vacuolation. Combination ERT and SRT decreased vacuolation to a greater extent, but still did not normalize morphology to that seen in age-matched wild-type mice. **Figure 7** (right panel) compares the quantitative analysis of DRG vacuolation resulting from the different treatment regimes. In untreated animals, the degree of DRG vacuolation approached 75%, *i.e.* three of every four DRG neurons were vacuolated. Although both ERT and SRT monotherapies decreased DRG vacuolation relative to that seen in untreated mice, the combination of ERT and SRT produced the largest beneficial effect, decreasing vacuolation by ∼50%. The reductions in all treatment groups were significant relative to the untreated Fabry-Rag control. Comparison of the ERT only treated group with the combination bi-monthly ERT with SRT (ERT2+SRT) showed significant benefit of the combined therapy approach. There was no significant difference between the ERT alone and the ERT every 4 months plus SRT (ERT4+SRT) indicating that for DRG vacuolation these treatments are equally effective. Collectively, these results imply that any aspect of Fabry peripheral neuropathy that may have been derived from DRG pathology (*e.g.* acroparesthesias, neuropathic pain) might be mitigated by a combined ERT plus SRT approach.

## Discussion

Clinical experience with ERT using recombinant human α-galactosidase A has shown it to be an effective therapy for many Fabry patients. Treatment results in positive effects on the heart, kidneys, peripheral nervous system and quality of life [34]. There is, however, evidence that ERT could be improved upon. For example, enzyme replacement with α-gal is unable to remove GL-3 from all tissues to the same degree. Endothelial cell clearance is much better than clearance from kidney podocytes, other epithelial cells, smooth muscle cells and cardiac myocytes [8], [35]. This is possibly due to restricted accessibility of the protein to these cell types. Given these considerations, alternative or additional therapeutic options could prove beneficial for Fabry patients.

A SRT approach has proven effective for Gaucher disease [11]. Because the pharmacodynamics and biodistribution of a small-molecule drug might differ from that of an enzyme, a SRT approach might provide benefit in tissues less accessible to ERT. The different functional modality of these two approaches was demonstrated by their differential effects on the lipid composition of the caveolae of vascular endothelium from Fabry mice [36]. However, as most male Fabry patients have essentially no α-gal enzyme activity, a SRT approach is unlikely to be effective as a monotherapy. This point was demonstrated in our studies evaluating SRT monotherapy using eliglustat tartrate in the Fabry mouse, in which a reduction in the rate of accumulation was achieved, but the GL-3 substrate continued to increase over time (Figure 1). This is in contrast to observations using SRT for type 1 Gaucher disease in which there are significant levels of residual enzyme activity and a monotherapy could successfully prevent or reverse disease progression [11], [19]. We went on to demonstrate that the most effective treatment at reducing the levels of GL-3 was by combining ERT every two months with SRT. This yielded the best efficacy at every time point in all tissues tested (Figure 4A) and was significantly better than ERT alone in the liver, kidney and urine. When comparing ERT alone to the ERT every two months plus SRT, significant improvements were also observed in urine uromodulin levels (Figure 5), loss of heat-sensitivity (Figure 6) and prevention of DRG cell vacuolation (Figure 7). These therapeutic improvements in divergent systems suggest that the combination therapy may be both additive and complementary.

As well as just adding SRT to the existing ERT, we evaluated the potential for a reduced frequency of ERT in combination with SRT (ERT4+SRT) as being another treatment option. In some assays, notably kidney and urine GL-3 levels (Figure 4), urine uromodulin level (Figure 5), heat-sensitivity (Figure 6) and degree of DRG vacuolation (Figure 7), this therapeutic option was as, or more effective than, ERT every two months alone. These results suggest that by using SRT the frequency of ERT infusions could be reduced, improving quality of life while maintaining therapeutic efficacy.

Although Fabry disease is caused by an X-linked mutation and therefore is often considered a predominantly male disorder, through lyonization there are many female Fabry heterozygotes that are also symptomatic [2], [37], [38]. Although the degree of disease severity tends to be milder and onset of disease symptoms delayed relative to males, there are female patients who are severely affected. These patients, who are already on ERT, would likely also benefit from the combination enzyme replacement and substrate reduction therapy. However, there is a subset of female Fabry patients whose disease symptoms are relatively mild or more slowly progressing who currently do not receive ERT. The option of a SRT alone may provide a realistic treatment alternative for these mildly affected patients. The SRT monotherapy studies in Fabry mice reported here provide evidence that therapeutic benefit might be attained through this approach. Similar effects were achieved in the preventative study (starting at one month of age) and the treatment study (starting at 3 months of age) with SRT alone, thus suggesting potential benefits for symptomatic patients. Furthermore, as mildly affected female Fabry patients express α-gal in a proportion of their cells, this might allow for some degree of enzyme cross-correction and render them amenable to an SRT monotherapy approach, *i.e.* similar to a type 1 Gaucher patient.

Conceptually, some symptoms of Fabry disease are unlikely to be treated effectively with ERT due to limited access of the protein therapeutic. These could include disease manifestations that may be related to the peripheral or central nervous systems, such as acroparesthesia, gastrointestinal distress, and psychological aspects [39] as well as hearing deficiencies and corneal opacity. A small molecule SRT-based treatment option may provide more benefit for such manifestations, as evidenced by the delayed loss of heat-sensitivity of Fabry-Rag mice treated with SRT (Figure 6). A second-generation molecule with properties similar to those of eliglustat tartrate, but able to cross the blood-brain barrier, may prove efficacious for treating eye, ear and CNS deficiencies.

In summary, our preclinical results in mouse models of Fabry disease suggest that for α-galactosidase A null Fabry patients, SRT as a monotherapy is unlikely to correct disease symptoms effectively. If this approach is combined with ERT, two new viable treatment options become possible. One allows for a reduced frequency of ERT while on SRT maintenance therapy, improving quality of life through a reduced dependency on enzyme infusions. The second approach adds SRT to the existing ERT, perhaps resulting in a treatment modality that is both additive and complementary with a more beneficial outcome than either treatment alone. For the currently under-treated mildly-affected female population, SRT alone may provide therapeutic benefit, as has been demonstrated for type 1 Gaucher patients. With carefully designed treatment regimens, adding SRT to the medical options for all Fabry patients could provide significant therapeutic and quality-of-life benefits.

## Materials and Methods

### Animal Studies

#### Ethics Statement

Procedures involving mice were reviewed and approved by the Genzyme Corporation Institutional Animal Care and Use Committee (Protocol 07-1115-2-BC) following guidelines established by the Association for Assessment of Accreditation of Laboratory Animal Care (AAALAC).

Wild-type 129/Sv mice were obtained from Taconic Laboratories (Germantown, NY). Fabry mice (α-galactosidase A knock-out) [21] were contract bred at Charles River Labs (Bedford, MA). Fabry-Rag mice are a stable cross of a Fabry mouse and a Rag-1 mouse [40] to generate an immune-deficient strain of mouse that accumulates GL-3 and can receive repeat doses of recombinant human α-galactosidase without developing a humoral immune response. Pathological and glycosphingolipid characterization revealed no discernible differences to the parental Fabry mouse line (data not shown), though differences in the rate of loss of heat-sensitivity (measured in the hot-plate assay – see below) were observed.

Urine was collected every two months. Mice were housed individually in metabolic cages for 24 h with unrestricted access to food and water. Blood samples were collected from the orbital venous plexus under anesthesia (2–3% isoflurane) into EDTA microhematocrit capillary tubes. Animals were killed by carbon dioxide inhalation, their tissues harvested immediately and snap frozen on dry ice. Spinal columns were taken whole and fixed in 10% neutral buffered formalin before processing for histological analysis.

### Treatment regimens

Animals in SRT treatment groups received eliglustat tartrate (Genz-112638; (1*R*,2*R*)-octanoic acid [2-(2,3-dihydro-benzo[1,4] dioxin- 6-yl)-2-hydroxy-1-pyrrolidin-1-ylmethyl -ethyl]-amide-L-tartaric acid salt) as a component of the pellet food diet. Drug was formulated at 0.15% w/w in standard mouse chow (TestDiet, Richmond, IN) and provided *ad libitum*. This formulation provided 300 mg/kg of eliglustat tartrate per day for a 25 g mouse eating 5 g of food per day. This dose of eliglustat tartrate was selected based on earlier pilot tolerability and efficacy studies (data not shown). Mice receiving recombinant human α-galactosidase A (1 mg/kg) were dosed by tail-vein injection. Mice dosed with α-gal every 2 months received injections at 3-, 5-, 7- and 9-months of age; the cohort receiving α-gal every 4 months was dosed at 3- and 7-months of age. Samples were collected prior to dosing.

### Measurement of peripheral sensory function using the hot-plate test

A nociceptive response to a heat stimulus was measured as described previously [23]. Mice were individually placed on a 55°C surface (Analgesia meter, Columbus Instruments, Columbus, OH) and the time taken to respond with a characteristic hind paw shake was recorded as the latency. If no response was evident by 60 sec the mouse was removed to prevent injury. The assay was performed every two months to minimize any potential learning effect.

### Quantitation of globotriaosylceramide (GL-3) levels

#### Tissue

On the day of extraction, tissues were removed from the −80°C freezer and placed on dry ice. Pieces of tissue were cut and placed into 20 mL glass vials and 1 mL of extraction solvent (80% methanol, 15% acetonitrile, 4% 10mM ammonium acetate solution, 1% formic acid) was added per 2.5 mg of tissue. Tissues were then homogenized (TissueTearor, BioSpec Products, Inc., Bartlesville, OK) and sonicated for 10 min at room temperature in a sonicating water bath. The resulting suspension was transferred to a 15 mL conical tube and the debris pelleted by centrifugation at 1500g for 5 min. A 4 mL aliquot of the supernatant was then transferred to an autosampler vial containing dried internal standard (N-heptadecanoyl ceramide trihexoside). The resulting solutions were vortexed for 5 min to ensure dissolution prior to analysis.

#### Urine

On the day of extraction, urine samples were removed from the −80°C freezer and allowed to thaw. An internal standard (N-heptadecanoyl ceramide trihexoside) was added to microcentrifuge tubes and dried under nitrogen gas. Urine (20 µl) was placed into the tubes, and vortexed 30 sec to solubilize the internal standard. Protein was precipitated from the sample by adding 100µl extraction solvent followed by sonication (sonicating waterbath for 10 min) and centrifugation (16,200g for 5 min) and an aliquot of the supernatant was then transferred to an autosampler vial for analysis.

#### Dried-Blood Spots

On the day of extraction, samples were removed from a −20°C freezer and allowed to come to room temperature. An internal standard (N-heptadecanoyl ceramide trihexoside) was added to microcentrifuge tubes and dried under nitrogen gas. A single 3.2 mm spot was punched from each card into a tube containing the dried internal standard. Protein was precipitated from the sample by adding 200µl extraction solvent followed by sonication (sonicating waterbath for 10 min) and centrifugation (16,200g for 5 min). An aliquot of the supernatant was then transferred to an autosampler vial for analysis.

#### LC/MS/MS

Samples were analyzed on a system consisting of an HTC PAL autosampler, Agilent 1200 HPLC, and API-5000 and 4000 Qtrap mass spectrometer. During analysis, samples were stored at 9°C in the autosampler cool stack. The HPLC was run in isocratic mode with a normal-phase silica column, and MS/MS was performed in MRM mode. Matrix based calibration curves were prepared to quantify GL-3 concentrations in urine, DBS and tissue.

### Quantitation of urine uromodulin

A sandwich ELISA was developed using commercially available reagents to quantitate uromodulin levels in the urine. The capture antibody was a goat polyclonal against an internal region of mouse Tamm-Horsfall protein (G-20; Santa Cruz Biotechnology, Inc., Santa Cruz, CA) and the detection antibody was the biotinylated sheep polyclonal anti-mouse uromodulin antibody (Cat# BAF5175, R&D Systems, Inc., Minneapolis, MN). Following incubation with streptavidin-HRP (Cat# 21127, Thermo Scientific, Rockland, IL) signal was detected using TMB HRP substrate (BioFX Laboratories, Inc., Owings Mills, MD).

### Histopathology

At the termination of the study, mice were killed by carbon dioxide asphyxiation. Whole spinal columns were fixed in 10% neutral buffered formalin and subsequently decalcified using buffered formic acid for 5–7 days. Decalcified spinal columns containing the spinal cord and DRGs were embedded in paraffin and 5–6 micron thick cross sections were stained with hematoxylin and eosin (H&E). A board-certified veterinary pathologist who was blinded to the study design examined multiple sections of spinal column including spinal cord and DRGs microscopically. For each section, the total number of normal or enlarged/vacuolated DRG cells was manually counted in a representative microscopic field at ×400 magnification. For quantitative assessment, the percent vacuolated DRG cells in each section were calculated by dividing the number of vacuolated DRG cells by the total number of DRG cells.
